# Correction: Patient education materials for non-specific low back pain and sciatica: A systematic review and meta-analysis

**DOI:** 10.1371/journal.pone.0307675

**Published:** 2024-07-18

**Authors:** Bradley Furlong, Holly Etchegary, Kris Aubrey-Bassler, Michelle Swab, Andrea Pike, Amanda Hall

S3 File is uploaded incorrectly. Please view the correct S3 File below.

## Supporting information

S3 File Summary of findings (all outcomes and comparisons). (DOCX)
