# Supplementary material for: Patient education materials for non-specific low back pain and sciatica: A systematic review and meta-analysis
Source: PLoS One. 2022 Oct 12;17(10):e0274527. doi: 10.1371/journal.pone.0274527 (PMC9555681; doi:10.1371/journal.pone.0274527)
Supplement: S1 File — (DOC) [file pone.0274527.s001.doc]

**Database: Ovid MEDLINE(R) and Epub Ahead of Print, In-Process, In-Data-Review & Other Non-Indexed Citations and Daily <1946 to March 24, 2022>**

Search Strategy:

--------------------------------------------------------------------------------

1 Back Pain/ (18402)

2 Low Back Pain/ (24663)

3 Sciatica/ (5132)

4 exp Spondylosis/ (8214)

5 back pain.ti,ab. (52845)

6 back ache.ti,ab. (111)

7 backache.ti,ab. (2587)

8 lumbar pain.ti,ab. (1647)

9 spine pain.ti,ab. (488)

10 spinal pain.ti,ab. (1644)

11 sciatica.ti,ab. (4587)

12 sciatic pain.ti,ab. (594)

13 spondylosis.ti,ab. (3517)

14 spondyloarthr*.ti,ab. (7933)

15 spondylolisthesis.ti,ab. (5635)

16 lumbago.ti,ab. (1408)

17 dorsalgia.ti,ab. (107)

18 or/1-17 (93541)

19 exp Health Education/ (257076)

20 exp Communications Media/ (366614)

21 Social Media/ (12867)

22 Internet/ (78659)

23 Mobile applications/ (9696)

24 Internet-Based Intervention/ (889)

25 exp Counseling/ (47228)

26 ed.fs. (294215)

27 education*.ti,ab. (619610)

28 psychoeducation*.ti,ab. (5995)

29 back school*.ti,ab. (304)

30 book*.ti,ab. (37582)

31 workbook*.ti,ab. (812)

32 (video or videos).ti,ab. (118444)

33 (audio or audiovisual*).ti,ab. (25653)

34 pamphlet*.ti,ab. (2127)

35 leaflet*.ti,ab. (24353)

36 brochure*.ti,ab. (2570)

37 (poster or posters).ti,ab. (7881)

38 (website* or web sites*).ti,ab. (36982)

39 (app or apps).ti,ab. (36123)

40 (application* adj2 (web or internet or online or mhealth or ehealth or digital or smartphone or cellphone or phone or ipad or iphone or android or mobile)).ti,ab. (15794)

41 infographic*.ti,ab. (697)

42 module*.ti,ab. (80774)

43 animation*.ti,ab. (3370)

44 ((patient or consumer or health) adj information).ti,ab. (34919)

45 ((biopsychosocial or psychosocial or psycho social or cognitive or behavioral or behavioural or psychological) adj2 (treatment* or intervention* or therapy or therapies or management or program* or training or approach* or counsel* or coach*)).ti,ab. (94595)

46 ((online or web or internet or e learning or elearning or ehealth or e health or telehealth or telephone or phone) adj2 (session* or program* or workshop* or training or coach* or counsel* or support*)).ti,ab. (15121)

47 ((group or individual or individuali* or personal or personali* or self) adj2 (session* or program* or workshop* or training or coach* or counsel* or support*)).ti,ab. (65872)

48 ((health or movement) adj coach*).ti,ab. (1048)

49 advice.ti,ab. (54323)

50 reassurance.ti,ab. (6430)

51 or/19-50 (1849755)

52 ((randomized controlled trial or controlled clinical trial).pt. or randomized.ab. or randomised.ab. or placebo.ab. or drug therapy.fs. or randomly.ab. or trial.ab. or groups.ab.) not (exp animals/ not humans.sh.) (4626609)

53 18 and 51 and 52 (2912)

***************************
