# Supplementary material for: Patient education materials for non-specific low back pain and sciatica: A systematic review and meta-analysis
Source: PLoS One. 2022 Oct 12;17(10):e0274527. doi: 10.1371/journal.pone.0274527 (PMC9555681; doi:10.1371/journal.pone.0274527)
Supplement: S2 File — (DOCX) [file pone.0274527.s002.docx]

**S2 File. Inclusion, exclusion, and GRADE criteria, protocol deviations**

# **Inclusion and exclusion criteria**

|  | **Inclusion criteria** | **Exclusion criteria** |
| --- | --- | --- |
| **Language** | Any language | - |
| **Study design** | Randomized controlled trials (RCTs) were included. Grey literature (e.g., theses), and pilot and feasibility studies were included so long as participants were randomly allocated to intervention and control groups. | All other non-RCT study designs. |
| **Population** | Adults aged 16 years or older with acute, subacute, or chronic non-specific LBP or sciatica. Our definition of non-specific LBP included populations with and without leg pain, but without nerve root compromise, as well as conditions such as spondylitis, spondylolysis, spondylolisthesis, disc protrusion, herniation or prolapse, and radicular syndrome. Sciatica was defined as pain radiating downwards from the buttock due to pressure on the lumbosacral nerve root. This nerve root compromise could involve inflammation or other immunological processes. | Subjects with specific pathology such as cauda equina syndrome, infection, neoplasm, fracture, or inflammatory disease, or if a large portion of the included participants were pregnant or had spinal surgery in the previous 12 months. There were no exclusion criteria based on care-seeking and non-care-seeking populations, so participants could be recruited either through physicians in a general practice setting or through the community (e.g., newspaper ads, online websites). |
| **Intervention** | Studies investigating the effect of patient education materials for LBP were included. Specifically, patient education materials were defined as interventions where any information about non-specific LBP or sciatica (e.g., diagnosis, prognosis, self-management or other treatment advice) was provided to the patient with a standardized evidence-based supplement (e.g., structured pamphlets, booklets, links to online resources, audio files, videos, or workbooks provided to the patient by a physician or member of the research team conducting the study. Education materials could be provided in person, via mail, or online. | Education delivered by other health professionals (e.g., chiropractors, physiotherapists) were not included. Studies where the education was solely aimed at teaching subjects how to perform exercises, or where the education was provided verbally from the physician or researcher without an evidence-based supplement were not included. Education materials are often provided as one component in a larger multi-component intervention; for this review, we were interested in interventions in which the educational material is the main component of the intervention. Therefore, interventions that include education materials plus another conservative component such as physiotherapy were excluded unless the comparison group allowed us to isolate the effect of the education material. |
| **Comparison** | We considered the effect of education materials compared to two main comparison groups (i) no other intervention and (ii) another conservative intervention. In cases where education was part of a multi-component intervention and was not the main component, they were included if the effect of the education alone could be determined (i.e., education + other conservative components vs. the conservative components alone which allows for determining the additive effect of education). | Comparisons of non-conservative treatments (e.g., spinal cord stimulations or surgery) were excluded. |
| **Outcomes** | We included process outcomes (the variables that are directly targeted by the education intervention and are thought to influence the clinical outcomes such as knowledge, pain self-efficacy, reassurance, pain-related anxiety, depression, and coping), clinical outcomes (those relevant to patients with low back pain including measures of pain, physical disability, and quality of life) and health-system outcomes (healthcare utilization measures like physician visits and imaging, and cost effectiveness) | We excluded a select few outcomes including flexibility and balance |

# **Grades of Recommendation, Assessment, Development and Evaluation approach (GRADE)**

GRADE involves assessing each study using five domains, each of which are "downgraded" a level of evidence if they meet the following criteria:

1. Quality - studies with high risk of bias contain greater than 25% of all participants

2. Inconsistency - high heterogeneity is clear from visual inspection or I²>75%

3. Indirectness - over 50% of participants are not in the target group (i.e., if participants were subject to multicomponent interventions where the effect of education alone may not be interpretable)

4. Imprecision - the comparison for continuous data involves less than 400 participants, or there are less than 300 events for dichotomous data

5. Publication bias - (i) many included studies have a small sample size, (ii) studies are or are likely to be industry-sponsored, (iii) other conflicts of interest are present. Publication bias was also assessed from visual inspection of a funnel plot if the analysis included 10 or more studies, as recommended in the Cochrane handbook. The treatment effect from each study was plotted against the sample size of each study. If the plot did not resemble a cone, or if the regression line was not perpendicular to the x axis, there may have been publication bias. If any of these criteria were present, we considered downgrading the quality of evidence of studies.

Studies were considered to have high quality evidence, moderate quality evidence, low quality evidence, very low-quality evidence, or no evidence if there were zero to four downgrades, respectively. When there was only one study in a comparison, it automatically received a very low-quality evidence assessment unless it was a large trial (n > 1000) with low risk of bias. In these cases, we would upgrade the assessment to low-quality evidence. For comparisons with studies that did not provide usable data for the meta-analysis (e.g., absence of summary data that we could not obtain after contacting authors), we provided a narrative synthesis of the studies alongside the analysis, and these studies were not included in GRADE assessment.

# **Protocol deviations**

## **Modifications to inclusion and exclusion criteria:**

In our initial literature screening, we found few studies (n = 9) where a physician provided the education material in a primary care or emergency department setting. We expanded these criteria to include studies where a member of the study’s research team could provide the education materials, rather than restricting this responsibility to a physician. Intuitively, to capture studies where PEMs were provided by a researcher, we allowed for inclusion of studies where participants were recruited outside of primary care or emergency department settings (e.g., through the community using online advertisements or local posters).

If a study’s inclusion/exclusion criteria did not match with our criteria of acute/subacute or chronic LBP, we went by the authors’ definition of LBP and included these studies in the respective analysis category. Where the authors did not specify the population as acute/subacute or chronic, we looked at the baseline demographic data to see if a distribution of LBP duration was provided. If the majority (>50%) of subjects had acute/subacute or chronic LBP, we included the study in the respective analysis category and accounted for this decision in our GRADE judgements (i.e., if more than half of the sample came from studies with unclear or mixed populations, we downgraded the quality of evidence for indirectness). There was a special case where a study included patients with a LBP duration of 6 weeks or greater and did not explicitly define their population as acute, subacute, or chronic. This fell between our two defined populations, but we included this study in the chronic LBP comparison because there would be no typical acute (< 6 weeks) LBP patients and we assumed there would be more chronic than subacute LBP patients due to the nature of these definitions. We accounted for this decision in our GRADE judgements of indirectness.

## **Modifications to the data synthesis:**

We originally planned to perform separate analyses for short (less than 6 months) and long-term (6 months or greater) follow-up time periods, but many studies had more than one follow-up during these timeframes. To better conform to the many follow-up time points and provide a more accurate depiction of how PEMs are effective over time, we included two additional time points (for a total of four) as described in our manuscript.

## **Modifications to sensitivity and subgroup analyses:**

We had planned to perform subgroup analyses on nonspecific LBP, sciatica, and mixed LBP populations based on exclusion or inclusion of patients with nerve root compromise, however, we found that in many studies this criterion was not specified, and no studies specifically stated they only included subjects with nerve root compromise. We therefore assumed the sciatica population to be minimal in included studies and considered all studies to have a population of non-specific LBP, so we could not perform this subgroup analysis. We also planned to perform subgroup analyses on hard vs. soft copy PEMs. Unfortunately, few studies used soft copy PEMs and most that did were isolated to one comparison. That is, four of five studies comparing PEMs to usual care for chronic LBP used soft copy PEMs, whereas only one study comparing PEMs to usual care for acute LBP used a soft copy PEM. Due to this imbalance between groups in the different comparisons, we did not see the value in performing this subgroup analysis.
