# Supplementary material for: Patient education materials for non-specific low back pain and sciatica: A systematic review and meta-analysis
Source: PLoS One. 2022 Oct 12;17(10):e0274527. doi: 10.1371/journal.pone.0274527 (PMC9555681; doi:10.1371/journal.pone.0274527)
Supplement: S3 File — (DOCX) [file pone.0274527.s003.docx]

**Legend: Non-Abbreviated Outcome Measures**

- **4DSQ:** Four-Dimensional Symptom Questionnaire
- **ADLQ:** Activities of Daily Living Questionnaire
- **ALBDS:** Aberdeen Pain and Function Scale
- **AQoL-8D:** Assessment of Quality of Life 8-Dimension
- **BPI:** Brief Pain Inventory
- **CSQ:** Coping Strategies Questionnaire
- **CPCI-42:** 42-Item Chronic Pain Coping Inventory
- **Dartmouth CO-OP:** Dartmouth Primary Care Cooperative Information Project
- **DASS-21:** 21-Item Depression Anxiety Stress Scale
- **EQ-5D:** the EuroQol 5-dimension health-related quality of life instrument
- **EQ5D-3L:** the EuroQol 5-dimension, 3-level health-related quality of life instrument
- **FABQ:** Fear-avoidance beliefs questionnaire
- **FFbH-R:** Hannover Functional Ability Questionnaire
- **GPE:** Global Perceived Effect scale
- **HAD:** Hospital Anxiety and Depression scale
- **NRS:** Numeric Rating Scale
- **ODI:** Oswestry Disability Index
- **OEQ:** Outcome Evaluation Questionnaire
- **PCS:** Pain Catastophizing Scale
- **PGIC:** Patients Global Impression of Change scale
- **PHQ-8:** 8-item Patient Health Questionnaire
- **PPQ:** Patient Pain Questionnaire
- **PSEQ:** Pain Self-Efficacy Questionnaire
- **PSEQ-2:** 2-Item Pain Self-Efficacy Questionnaire
- **PSS:** Perceived Stress Scale
- **QBPDS:** Quebec Back Pain Disability Scale
- **RMDQ:** Roland Morris Disability Questionnaire
- **SAS:** Zung Self-Rating Anxiety Scale
- **SBS:** Symptom Bothersomeness scale
- **SDS:** Zung Self-Rating Depression Scale
- **SF-12:** 12-Item Short Form Survey
- **SF-36:** 36-Item Short Form Survey
- **TSK-4:** 4-item Tampa Scale for Kinesiophobia
- **UTs:** unvalidated tools (unspecified, bespoke, or unnecessary adaptations of already validated tools with insufficient information to determine their validity)
- **VAS:** Visual Analogue Scale
- **VNS:** Visual Numeric Scale
- **WLQ:** Work Limitations Questionnaire

| Education materials compared with no intervention (usual care) for acute/subacute low back pain  P: adults aged 16+ with acute/subacute low back pain (<12 weeks duration)  I: education materials (typically provided during a single encounter with a physician or researcher in-person, via parcel, or over the internet)  C: no intervention or usual care | | | | |
| --- | --- | --- | --- | --- |
| Outcome (# studies)  Time points | **Outcome measurement tools^a^** | **SMD^b^ (95% CI) or RR^+,-^ (95% CI)** | **Participants**  **(# studies)** | **Quality of Evidence^c^ (GRADE)** |
| Knowledge (n = 5): | | | | |
| - Immediate-term (1-8 wks) | UTs (4) | -0.51 [-0.72, -0.31] | 699 (4) | ⊕⊕⊖⊖ Low^1,4*^ |
| - Short-term (13-16 wks) | UTs (2) | -0.48 [-0.90, -0.05] | 502 (2) | ⊕⊕⊖⊖ Low^1,4*^ |
| - Medium-term | - | - | 0 (0) | No evidence |
| - Long-term (52 wks) | UTs (1) | RR^+^ = 1.28 [1.10, 1.49] | 777 (1) | ⊕⊖⊖⊖ Very low^6^ |
| Self-efficacy (n = 4): | | | | |
| - Immediate-term (2-8 wks) | PSEQ-2 (1), UTs (3) | -0.28 [-0.63, 0.07] | 650 (3) | ⊕⊕⊕⊖ Moderate^4*^ |
| - Short-term (16 wks) | UTs (1) | -0.78 [-0.98, -0.58] | 398 (1) | ⊕⊖⊖⊖ Very low^6^ |
| - Medium-term | - | - | 0 (0) | No evidence |
| - Long-term (52 wks) | UTs (1) | -0.32 [-0.52, -0.12] | 421 (1) | ⊕⊖⊖⊖ Very low^6^ |
| Attitudes: no evidence | | | | |
| General beliefs: no evidence | | | | |
| Fear-avoidance (n = 3): | | | | |
| - Immediate-term (1-6 wks) | FABQ (2), UTs (1) | -0.14 [-0.36, 0.09] | 611 (3) | ⊕⊕⊕⊕ High |
| - Short-term (13 wks) | FABQ (1) | 0.00 [-0.38, 0.38] | 114 (1) | ⊕⊖⊖⊖ Very low^6^ |
| - Medium-term | - | - | 0 (0) | No evidence |
| - Long-term (52 wks) | FABQ (1) | 0.10 [-0.15, 0.35] | 150 (1) | ⊕⊖⊖⊖ Very low^6^ |
| Catastrophizing (n = 3): | | | | |
| - Immediate-term (2-8 wks) | TSK-4 (1), CSQ (1), UTs (1) | -0.01 [-0.22, 0.20] | 879 (3) | ⊕⊕⊕⊕ High |
| - Short-term (16 wks) | TSK-4 (1) | -0.12 [-0.31, 0.07] | 398 (1) | ⊕⊖⊖⊖ Very low^6^ |
| - Medium-term | - | - | 0 (0) | No evidence |
| - Long-term (52 wks) | CSQ (1) | 0.07 [-0.18, 0.32] | 248 (1) | ⊕⊖⊖⊖ Very low^6^ |
| Coping: no evidence | | | | |
| Anxiety (n = 3): | | | | |
| - Immediate-term (2 wks) | 4DSQ (1), UTs (1) | -0.01 [-0.45, 0.43] | 485 (2) | ⊕⊕⊕⊖ Moderate^3^ |
| - Short-term | - | - | 0 (0) | No evidence |
| - Medium-term | - | - | 0 (0) | No evidence |
| - Long-term (52 wks) | 4DSQ (1), UTs (1) | -0.13 [-0.52, 0.26] | 673 (2) | ⊕⊕⊖⊖ Low^1,3^ |
| Stress: no evidence | | | | |
| Depression: no evidence | | | | |
| Pain (n = 5): | | | | |
| - Immediate-term (2-8 wks) | NRS (2), UTs (1) | -0.13 [-0.27, 0.01] | 910 (3) | ⊕⊕⊕⊕ High |
| - Short-term (12-16 wks) | NRS (3), UTs (1) | -0.24 [-0.42, -0.06] | 1101 (4) | ⊕⊕⊕⊕ High |
| - Medium-term (26 wks) | NRS (2) | -0.03 [-0.20, 0.15] | 515 (2) | ⊕⊕⊕⊕ High |
| - Long-term (52 wks) | NRS (2), VNS (1) | -0.11 [-0.24, 0.02] | 892 (3) | ⊕⊕⊕⊖ Moderate^1^ |
| Disability (n = 8): | | | | |
| - Immediate-term (1-8 wks) | RMDQ (2), ALBDS (2), FFbH-R (1), WLQ (1) | -0.05 [-0.17, 0.06] | 1220 (6) | ⊕⊕⊕⊕ High |
| - Short-term (13-16 wks) | RMDQ (2), ALBDS (1), FFbH-R (1), WLQ (1), ODI (1) | -0.06 [-0.18, 0.05] | 1272 (6) | ⊕⊕⊕⊕ High |
| - Medium-term (26 wks) | RMDQ (2), ALBDS (1) | 0.09 [-0.08, 0.27] | 563 (3) | ⊕⊕⊕⊕ High |
| - Long-term (52 wks) | RMDQ (2), ALBDS (1), ODI (1) | -0.09 [-0.27, 0.08] | 938 (4) | ⊕⊕⊕⊖ Moderate^1^ |
| Quality of Life (n = 4): | | | | |
| - Immediate-term (1-8 wks) | SF-36 (1), Dartmouth CO-OP (1) | -0.24 [-0.42, -0.07] | 524 (2) | ⊕⊕⊕⊖ Moderate^4*^ |
| - Short-term (13-16 wks) | SF-36 (1), Dartmouth CO-OP (1), UTs (1) | -0.20 [-0.43, 0.03] | 804 (3) | ⊕⊕⊕⊕ High |
| - Medium-term (26 wks) | UTs (1) | 0.00 [-0.23, 0.23] | 286 (1) | ⊕⊖⊖⊖ Very low^6^ |
| - Long-term (52 wks) | EQ5D-3L (1), UTs (1) | 0.01 [-0.17, 0.19] | 470 (2) | ⊕⊕⊕⊖ Moderate^1^ |
| Global improvement (n = 1): | | | | |
| - Immediate-term (6 wks) | UTs (1) | RR^-^ = 1.07 [0.80, 1.43] | 305 (1) | ⊕⊖⊖⊖ Very low^6^ |
| - Short-term (13 wks) | UTs (1) | RR^-^ = 1.03 [0.75, 1.42] | 305 (1) | ⊕⊖⊖⊖ Very low^6^ |
| - Medium-term (26 wks) | UTs (1) | RR^-^ = 1.05 [0.75, 1.47] | 299 (1) | ⊕⊖⊖⊖ Very low^6^ |
| - Long-term (52 wks) | UTs (1) | RR^-^ = 1.15 [0.81, 1.65] | 288 (1) | ⊕⊖⊖⊖ Very low^6^ |
| Function: no evidence | | | | |
| Days off work (n = 3): | | | | |
| - Immediate-term (6 wks) | % with days off work (1) | RR^-^ = 0.83 [0.49, 1.42] | 248 (1) | ⊕⊖⊖⊖ Very low^6^ |
| - Short-term (13 wks) | % with days off work (1), mean days off work (1) | -0.35 [-0.63, -0.08] | 612 (2) | ⊕⊕⊖⊖ Low^1,4*^ |
| - Medium-term (26 wks) | % with days off work (1) | RR^-^ = 0.33 [0.10, 1.16] | 244 (1) | ⊕⊖⊖⊖ Very low^6^ |
| - Long-term (52 wks) | % with days off work (1), mean days off work (2) | -0.10 [-0.32, 0.12] | 1535 (3) | ⊕⊕⊕⊖ Moderate^1^ |
| Imaging (n = 1): | | | | |
| - Immediate-term | - | - | 0 (0) | No evidence |
| - Short-term (13 wks) | % receiving LBP imaging (1) | RR^-^ = 0.64 [0.38, 1.09] | 364 (1) | ⊕⊖⊖⊖ Very low^6^ |
| - Medium-term | - | - | 0 (0) | No evidence |
| - Long-term (52 wks) | % receiving LBP imaging (1) | RR^-^ = 0.60 [0.41, 0.89] | 364 (1) | ⊕⊖⊖⊖ Very low^6^ |
| Physician visits (n = 3): | | | | |
| - Immediate-term | - | - | 0 (0) | No evidence |
| - Short-term (13 wks) | Mean physician visits (1) | -0.07 [-0.27, 0.13] | 364 (1) | ⊕⊖⊖⊖ Very low^6^ |
| - Medium-term | - | - | 0 (0) | No evidence |
| - Long-term (52 wks) | Mean physician visits (2), % with physician visit (1) | -0.16 [-0.26, -0.05] | 1721 (3) | ⊕⊕⊕⊖ Moderate^1^ |
| Referrals (n = 1): | | | | |
| - Immediate-term | - | - | 0 (0) | No evidence |
| - Short-term | - | - | 0 (0) | No evidence |
| - Medium-term | - | - | 0 (0) | No evidence |
| - Long-term (52 wks) | Proportion with specialist referral (1) | RR^-^ = 0.85 [0.58, 1.23] | 936 (1) | ⊕⊖⊖⊖ Very low^6^ |
| Cost (n = 1): | | | | |
| - Immediate-term | - | - | 0 (0) | No evidence |
| - Short-term | - | - | 0 (0) | No evidence |
| - Medium-term (26 wks) | Quality-adjusted life years (1) | -0.11 [-0.37, 0.16] | 226 (1) | ⊕⊖⊖⊖ Very low^6^ |
| - Long-term | - | - | 0 (0) | No evidence |

^a^See legend on first page of S3 File for non-abbreviated names of measurement tools. **^b^**Data are presented as standardized mean differences (SMD) and 95% confidence intervals (95% CI) unless otherwise indicated (negative SMD favors education materials). Risk ratios are indicated with RR^+^ (RR > 1 favors education) and RR^-^ (RR < 1 favors education). ^c^Quality of evidence was downgraded for risk of bias,**^1^** imprecision,**^2^** inconsistency,**^3^** indirectness,**^4^** publication bias,**^5^** or downgraded to very low if there was one study.**^6^** In this comparison, downgrades for risk of bias and inconsistency followed our pre-defined cut-offs (S2 File) and do not require further interpretation. *Where evidence for knowledge, pain self-efficacy, quality of life, and days off work were downgraded for indirectness, this was due to Irvine *et al.,* 2015 (they did not explicitly define their LBP population) or Simula *et al.,* 2021 (PEMs could be given by other providers and not just physicians, however, we decided to include this study since they provided a detailed breakdown of data for each provider and almost half of the sample saw a physician).

| Education materials compared with another intervention for acute/subacute low back pain  P: adults aged 16+ with acute/subacute low back pain (<12 weeks duration)  I: education materials (typically provided during a single encounter with a physician or researcher in-person, via parcel, or over the internet)  C: any non-conservative intervention (e.g., yoga, massage, exercise, cognitive behavioural therapy, etc.) | | | | |
| --- | --- | --- | --- | --- |
| Outcome (# studies)  Time points | **Outcome measurement tools^a^** | **SMD^b^ (95% CI) or RR^+,-^ (95% CI)** | **Participants**  **(# studies)** | **Quality of Evidence^c^ (GRADE)** |
| Knowledge: no evidence | | | | |
| Self-Efficacy: no evidence | | | | |
| Attitudes: no evidence | | | | |
| General beliefs: no evidence | | | | |
| Fear-Avoidance (n = 1): | | | | |
| - Immediate-term | - | - | 0 (0) | No evidence |
| - Short-term | - | - | 0 (0) | No evidence |
| - Medium-term | - | - | 0 (0) | No evidence |
| - Long-term (52 wks) | FABQ (1) | 0.17 [-0.16, 0.49] | 155 (1) | ⊕⊖⊖⊖ Very low^6^ |
| Catastrophizing (n = 1): | | | | |
| - Immediate-term | - | - | 0 (0) | No evidence |
| - Short-term | - | - | 0 (0) | No evidence |
| - Medium-term | - | - | 0 (0) | No evidence |
| - Long-term (52 wks) | PCS (1) | -0.06 [-0.38, 0.27] | 155 (1) | ⊕⊖⊖⊖ Very low^6^ |
| Coping: no evidence | | | | |
| Anxiety (n = 1): | | | | |
| - Immediate-term | - | - | 0 (0) | No evidence |
| - Short-term | - | - | 0 (0) | No evidence |
| - Medium-term | - | - | 0 (0) | No evidence |
| - Long-term (52 wks) | HAD (1) | -0.05 [-0.37, 0.27] | 155 (1) | ⊕⊖⊖⊖ Very low^6^ |
| Stress: no evidence | | | | |
| Depression (n = 1): | | | | |
| - Immediate-term | - | - | 0 (0) | No evidence |
| - Short-term | - | - | 0 (0) | No evidence |
| - Medium-term | - | - | 0 (0) | No evidence |
| - Long-term (52 wks) | HAD (1) | 0.00 [-0.32, 0.32] | 155 (1) | ⊕⊖⊖⊖ Very low^6^ |
| Pain (n = 3): | | | | |
| - Immediate-term (4 wks) | SBS (1) | 0.51 [0.20, 0.83] | 178 (1) | ⊕⊖⊖⊖ Very low^6^ |
| - Short-term (12 wks) | VAS (1), SBS (1) | 0.07 [-0.81, 0.95] | 212 (2) | ⊕⊕⊖⊖ Low^2,3^ |
| - Medium-term (26 wks) | VAS (1) | -0.89 [-1.66, -0.11] | 31 (1) | ⊕⊖⊖⊖ Very low^6^ |
| - Long-term (52 wks) | OEQ (1) | 0.04 [-0.28, 0.36] | 155 (1) | ⊕⊖⊖⊖ Very low^6^ |
| Disability (n = 3): | | | | |
| - Immediate-term (4 wks) | RMDQ (1) | 0.27 [-0.04, 0.58] | 178 (1) | ⊕⊖⊖⊖ Very low^6^ |
| - Short-term (12 wks) | RMDQ (2) | 0.23 [-0.06, 0.51] | 212 (2) | ⊕⊕⊕⊖ Moderate^2^ |
| - Medium-term (26 wks) | RMDQ (1) | -0.15 [-0.88, 0.58] | 31 (1) | ⊕⊖⊖⊖ Very low^6^ |
| - Long-term (48-52 wks) | ADLQ (1), % with reduced activity (1) | 0.20 [-0.04, 0.43] | 343 (2) | ⊕⊕⊖⊖ Low^2,4*^ |
| Quality of Life: no evidence | | | | |
| Global Improvement: no evidence | | | | |
| Function: no evidence | | | | |
| Days off work (n = 2): | | | | |
| - Immediate-term | - | - | 0 (0) | No evidence |
| - Short-term | - | - | 0 (0) | No evidence |
| - Medium-term | - | - | 0 (0) | No evidence |
| - Long-term (48-52 wks) | % with days off work (1), mean days off work (1) | 0.36 [0.09, 0.63] | 343 (2) | ⊕⊕⊖⊖ Low^2,4*^ |
| Imaging: no evidence | | | | |
| Physician visits (n = 1): | | | | |
| - Immediate-term | - | - | 0 (0) | No evidence |
| - Short-term | - | - | 0 (0) | No evidence |
| - Medium-term | - | - | 0 (0) | No evidence |
| - Long-term (52 wks) | Mean physician visits (1) | 0.53 [0.20, 0.85] | 155 (1) | ⊕⊖⊖⊖ Very low^6^ |
| Referrals: no evidence | | | | |
| Cost: no evidence | | | | |

^a^See legend on first page of S3 File for non-abbreviated names of measurement tools. **^b^**Data are presented as standardized mean differences (SMD) and 95% confidence intervals (95% CI) unless otherwise indicated (negative SMD favors education materials). Risk ratios are indicated with RR^+^ (RR > 1 favors education) and RR^-^ (RR < 1 favors education). ^c^Quality of evidence was downgraded for risk of bias,**^1^** imprecision,**^2^** inconsistency,**^3^** indirectness,**^4^** publication bias,**^5^** or downgraded to very low if there was one study.**^6^** In this comparison, downgrades for imprecision and inconsistency followed our pre-defined cut-offs (supplemental file X) and do not require further interpretation. *Where evidence for disability and days off work were downgraded for indirectness, this was due to Linton *et al.,* 2000 (did not explicitly define their LBP population) and Cherkin *et al.,* 1998, (mixed LBP population: 72% acute, 28% chronic).

| Education materials compared with no intervention (usual care) for chronic low back pain  P: adults aged 16+ with chronic low back pain (≥ 12 weeks duration)  I: education materials (typically provided during a single encounter with a physician or researcher in-person, via parcel, or over the internet)  C: no intervention or usual care | | | | |
| --- | --- | --- | --- | --- |
| Outcome (# studies)  Time points | **Outcome measurement tools^a^** | **SMD^b^ (95% CI) or RR^+,-^ (95% CI)** | **Participants**  **(# studies)** | **Quality of Evidence^c^ (GRADE)** |
| Knowledge: no evidence | | | | |
| Self-Efficacy (n = 1): | | | | |
| - Immediate (6 wks) | PSEQ (1) | -0.21 [-0.39, -0.03] | 461 (1) | ⊕⊖⊖⊖ Very low^6^ |
| - Short-term (13 wks) | PSEQ (1) | -0.25 [-0.43, -0.06] | 461 (1) | ⊕⊖⊖⊖ Very low^6^ |
| - Medium-term (26 wks) | PSEQ (1) | -0.23 [-0.41, -0.05] | 461 (1) | ⊕⊖⊖⊖ Very low^6^ |
| - Long-term (39 wks) | PSEQ (1) | -0.32 [-0.50, -0.13] | 461 (1) | ⊕⊖⊖⊖ Very low^6^ |
| Attitudes: no evidence | | | | |
| General beliefs: no evidence | | | | |
| Fear Avoidance (n = 2): | | | | |
| - Immediate (2-6 wks) | FABQ (2) | -0.15 [-0.33, 0.02] | 505 (2) | ⊕⊕⊕⊕ High |
| - Short-term (13 wks) | FABQ (1) | -0.09 [-0.27, 0.09] | 461 (1) | ⊕⊖⊖⊖ Very low^6^ |
| - Medium-term (26 wks) | FABQ (1) | -0.24 [-0.43, -0.06] | 461 (1) | ⊕⊖⊖⊖ Very low^6^ |
| - Long-term (39 wks) | FABQ (1) | -0.16 [-0.34, 0.02] | 461 (1) | ⊕⊖⊖⊖ Very low^6^ |
| Catastrophizing: no evidence | | | | |
| Coping: no evidence | | | | |
| Anxiety: no evidence | | | | |
| Stress (n = 1): | | | | |
| - Immediate (6 wks) | PSS (1) | -0.13 [-0.32, 0.05] | 461 (1) | ⊕⊖⊖⊖ Very low^6^ |
| - Short-term (13 wks) | PSS (1) | -0.13 [-0.31, 0.06] | 461 (1) | ⊕⊖⊖⊖ Very low^6^ |
| - Medium-term (26 wks) | PSS (1) | -0.15 [-0.33, 0.03] | 461 (1) | ⊕⊖⊖⊖ Very low^6^ |
| - Long-term (39 wks) | PSS (1) | -0.21 [-0.39, -0.03] | 461 (1) | ⊕⊖⊖⊖ Very low^6^ |
| Depression (n = 1): | | | | |
| - Immediate (6 wks) | PHQ-8 (1) | -0.18 [-0.36, 0.01] | 461 (1) | ⊕⊖⊖⊖ Very low^6^ |
| - Short-term (13 wks) | PHQ-8 (1) | -0.09 [-0.27, 0.09] | 461 (1) | ⊕⊖⊖⊖ Very low^6^ |
| - Medium-term (26 wks) | PHQ-8 (1) | -0.11 [-0.29, 0.07] | 461 (1) | ⊕⊖⊖⊖ Very low^6^ |
| - Long-term (39 wks) | PHQ-8 (1) | -0.15 [-0.33, 0.03] | 461 (1) | ⊕⊖⊖⊖ Very low^6^ |
| Pain (n = 5): | | | | |
| - Immediate (2-6 wks) | VAS (2), NRS (1), UTs (1) | -0.16 [-0.29, -0.03] | 890 (4) | ⊕⊕⊕⊖ Moderate^1^ |
| - Short-term (12-13 wks) | VAS (2), NRS (1), UTs (1) | -0.44 [-0.88, 0.00] | 925 (4) | ⊕⊕⊖⊖ Low^1,3^ |
| - Medium-term (24-26 wks) | VAS (2), NRS (1), UTs (1) | -0.53 [-1.01, -0.05] | 907 (4) | ⊕⊕⊖⊖ Low^1,3^ |
| - Long-term (39-52 wks) | VAS (1), NRS (1) | -0.21 [-0.41, -0.01] | 757 (2) | ⊕⊕⊕⊖ Moderate^1^ |
| Disability (n = 5): | | | | |
| - Immediate (2-6 wks) | RMDQ (4) | -0.12 [-0.31, 0.07] | 919 (4) | ⊕⊕⊕⊖ Moderate^1^ |
| - Short-term (12-13 wks) | RMDQ (3), QBPDS (1) | -0.23 [-0.48, 0.03] | 964 (4) | ⊕⊕⊕⊖ Moderate^1^ |
| - Medium-term (24-26 wks) | RMDQ (3), QBPDS (1) | -0.32 [-0.61, -0.03] | 939 (4) | ⊕⊕⊕⊖ Moderate^1^ |
| - Long-term (39-52 wks) | RMDQ (2) | -0.12 [-0.27, 0.02] | 770 (2) | ⊕⊕⊕⊖ Moderate^1^ |
| Quality of Life (n = 4): | | | | |
| - Immediate (4-6 wks) | AQoL-8D (1), SF-12 (1), EQ-5D (1) | -0.04 [-0.18, 0.09] | 839 (3) | ⊕⊕⊕⊖ Moderate^1^ |
| - Short-term (12-13 wks) | AQoL-8D (1), SF-12 (1), SF-36 (1), EQ-5D (1) | -0.15 [-0.28, -0.03] | 934 (4) | ⊕⊕⊕⊖ Moderate^1^ |
| - Medium-term (24-26 wks) | AQoL-8D (1), SF-12 (1), SF-36 (1), EQ-5D (1) | -0.23 [-0.41, -0.04] | 902 (4) | ⊕⊕⊕⊖ Moderate^1^ |
| - Long-term (39-52 wks) | AQoL-8D (1), EQ-5D (1) | -0.13 [-0.28, 0.01] | 748 (2) | ⊕⊕⊕⊖ Moderate^1^ |
| Global Improvement | | | | |
| - Immediate (6 wks) | GPE (1) | -0.40 [-0.58, -0.21] | 461 (1) | ⊕⊖⊖⊖ Very low^6^ |
| - Short-term (13 wks) | GPE (1) | -0.42 [-0.60, -0.24] | 461 (1) | ⊕⊖⊖⊖ Very low^6^ |
| - Medium-term (26 wks) | GPE (1) | -0.46 [-0.65, -0.28] | 461 (1) | ⊕⊖⊖⊖ Very low^6^ |
| - Long-term (39 wks) | GPE (1) | -0.43 [-0.61, -0.24] | 461 (1) | ⊕⊖⊖⊖ Very low^6^ |
| Function: no evidence | | | | |
| Days off work: no evidence | | | | |
| Imaging: no evidence | | | | |
| Physician Visits: no evidence | | | | |
| Referrals: no evidence | | | | |
| Cost: no evidence | | | | |

^a^See legend on first page of S3 File for non-abbreviated names of measurement tools. **^b^**Data are presented as standardized mean differences (SMD) and 95% confidence intervals (95% CI) unless otherwise indicated (negative SMD favors education materials). Risk ratios are indicated with RR^+^ (RR > 1 favors education) and RR^-^ (RR < 1 favors education). ^c^Quality of evidence was downgraded for risk of bias,**^1^** imprecision,**^2^** inconsistency,**^3^** indirectness,**^4^** publication bias,**^5^** or downgraded to very low if there was one study.**^6^** In this comparison, all downgrade decisions followed our pre-defined cut-offs (S2 File) and do not require further interpretation.

| Education materials compared with another intervention for chronic low back pain  P: adults aged 16+ with chronic low back pain (≥ 12 weeks duration)  I: education materials (typically provided during a single encounter with a physician or researcher in-person, via parcel, or over the internet)  C: any non-conservative intervention (e.g., yoga, massage, exercise, cognitive behavioural therapy, etc.) | | | | |
| --- | --- | --- | --- | --- |
| Outcome (# studies)  Time points | **Outcome measurement tools^a^** | **SMD^b^ (95% CI) or RR^+,-^ (95% CI)** | **Participants**  **(# studies)** | **Quality of Evidence^c^ (GRADE)** |
| Knowledge: no evidence | | | | |
| Self-Efficacy (n = 1): | | | | |
| - Immediate-term (4 wks) | PSEQ (1) | 0.05 [-0.23, 0.33] | 199 (1) | ⊕⊖⊖⊖ Very low^6^ |
| - Short-term (12 wks) | PSEQ (1) | 0.06 [-0.22, 0.34] | 199 (1) | ⊕⊖⊖⊖ Very low^6^ |
| - Medium-term (24 wks) | PSEQ (1) | 0.04 [-0.24, 0.32] | 199 (1) | ⊕⊖⊖⊖ Very low^6^ |
| - Long-term | - | - | 0 (0) | No evidence |
| Attitudes: no evidence | | | | |
| General beliefs: no evidence | | | | |
| Fear-Avoidance (n = 1): | | | | |
| - Immediate-term (4 wks) | FABQ (1) | 0.13 [-0.15, 0.41] | 199 (1) | ⊕⊖⊖⊖ Very low^6^ |
| - Short-term (12 wks) | FABQ (1) | 0.08 [-0.20, 0.36] | 199 (1) | ⊕⊖⊖⊖ Very low^6^ |
| - Medium-term (24 wks) | FABQ (1) | 0.00 [-0.28, 0.28] | 199 (1) | ⊕⊖⊖⊖ Very low^6^ |
| - Long-term | - | - | 0 (0) | No evidence |
| Catastrophizing (n = 1) | | | | |
| - Immediate-term (4 wks) | PCS (1) | 0.50 [0.21, 0.78] | 199 (1) | ⊕⊖⊖⊖ Very low^6^ |
| - Short-term (12 wks) | PCS (1) | 0.42 [0.14, 0.70] | 199 (1) | ⊕⊖⊖⊖ Very low^6^ |
| - Medium-term (24 wks) | PCS (1) | 0.44 [0.15, 0.72] | 199 (1) | ⊕⊖⊖⊖ Very low^6^ |
| - Long-term | - | - | 0 (0) | No evidence |
| Coping (n = 1): | | | | |
| - Immediate-term (4 wks) | CPCI-42 (1) | 0.13 [-0.14, 0.41] | 199 (1) | ⊕⊖⊖⊖ Very low^6^ |
| - Short-term (12 wks) | CPCI-42 (1) | 0.22 [-0.05, 0.50] | 199 (1) | ⊕⊖⊖⊖ Very low^6^ |
| - Medium-term (24 wks) | CPCI-42 (1) | 0.17 [-0.10, 0.45] | 199 (1) | ⊕⊖⊖⊖ Very low^6^ |
| - Long-term | - | - | 0 (0) | No evidence |
| Anxiety (n = 2): | | | | |
| - Immediate-term (4 wks) | DASS-21 (1) | 0.07 [-0.20, 0.35] | 199 (1) | ⊕⊖⊖⊖ Very low^6^ |
| - Short-term (9-12 wks) | DASS-21 (1), SAS (1) | 0.65 [-0.58, 1.87] | 229 (2) | ⊕⊕⊖⊖ Low^2,3^ |
| - Medium-term (24 wks) | DASS-21 (1) | 0.13 [-0.15, 0.40] | 199 (1) | ⊕⊖⊖⊖ Very low^6^ |
| - Long-term | - | - | 0 (0) | No evidence |
| Stress (n = 1): | | | | |
| - Immediate-term (4 wks) | DASS-21 (1) | 0.17 [-0.10, 0.45] | 199 (1) | ⊕⊖⊖⊖ Very low^6^ |
| - Short-term (12 wks) | DASS-21 (1) | 0.31 [0.03, 0.59] | 199 (1) | ⊕⊖⊖⊖ Very low^6^ |
| - Medium-term (24 wks) | DASS-21 (1) | 0.26 [-0.02, 0.54] | 199 (1) | ⊕⊖⊖⊖ Very low^6^ |
| - Long-term | - | - | 0 (0) | No evidence |
| Depression | | | | |
| - Immediate-term (4 wks) | DASS-21 (1) | 0.03 [-0.25, 0.31] | 199 (1) | ⊕⊖⊖⊖ Very low^6^ |
| - Short-term (9-12 wks) | DASS-21 (1), SDS (1) | 0.79 [-0.56, 2.14] | 229 (2) | ⊕⊕⊖⊖ Low^2,3^ |
| - Medium-term (24 wks) | DASS-21 (1) | 0.18 [-0.10, 0.46] | 199 (1) | ⊕⊖⊖⊖ Very low^6^ |
| - Long-term | - | - | 0 (0) | No evidence |
| Pain (n = 10): | | | | |
| - Immediate-term (4-8 wks) | SBS (3), VAS (1), NRS (1), BPI (1), PPQ (1), UTs (1) | 0.30 [0.03, 0.56] | 732 (8) | ⊕⊕⊕⊕ High |
| - Short-term (9-12 wks) | NRS (3), SBS (2), BPI (1), UTs (1) | 0.54 [0.20, 0.88] | 815 (7) | ⊕⊕⊕⊕ High* |
| - Medium-term (24-26 wks) | SBS (2), BPI (1), UTs (1) | 0.22 [-0.25, 0.69] | 450 (4) | ⊕⊕⊕⊖ Moderate^3^ |
| - Long-term (52 wks) | SBS (1) | 0.18 [-0.12, 0.48] | 168 (1) | ⊕⊖⊖⊖ Very low^6^ |
| Disability (n = 9): | | | | |
| - Immediate-term (4-8 wks) | RMDQ (6), ODI (1) | 0.47 [0.12, 0.83] | 714 (7) | ⊕⊕⊕⊕ High* |
| - Short-term (9-12 wks) | RMDQ (6), ODI (2) | 0.64 [0.25, 1.02] | 881 (8) | ⊕⊕⊕⊕ High* |
| - Medium-term (24-26 wks) | RMDQ (3), ODI (1) | 0.29 [-0.09, 0.67] | 450 (4) | ⊕⊕⊕⊕ High |
| - Long-term (52 wks) | RMDQ (1) | -0.07 [-0.37, 0.23] | 168 (1) | ⊕⊖⊖⊖ Very low^6^ |
| Quality of Life (n = 5): | | | | |
| - Immediate-term (4-8 wks) | SF-36 (3), SF-12 (1) | 1.25 [0.14, 2.36]  Two studies did not provide usable data but found no difference between groups | 62 (2)  221 (2) | ⊕⊕⊖⊖ Low^1,2^ |
| - Short-term (10-12 wks) | SF-36 (3), SF-12 (1) | 1.01 [-0.99, 3.01]  Two studies did not provide usable data but found (i) no difference between groups or (ii) education to be less effective than other interventions | 228 (2)  i. 66 (1)  ii. 168 (1) | ⊕⊕⊖⊖ Low^2,3^ |
| - Medium-term (26 wks) | SF-36 (1) | One study did not provide usable data but found no difference between groups | 63 (1) | ⊕⊖⊖⊖ Very low^6^ |
| - Long-term (52 wks) | SF-12 (1) | One study did not provide usable data but found no difference between groups | 159 (1) | ⊕⊖⊖⊖ Very low^6^ |
| Global Improvement (n = 3): | | | | |
| - Immediate-term (4-6 wks) | PGIC (1), UTs (1) | 0.53 [0.21, 0.84] | 327 (2) | ⊕⊕⊕⊖ Moderate^2^ |
| - Short-term (12 wks) | PGIC (1), UTs (2) | 0.60 [0.16, 1.04] | 509 (3) | ⊕⊕⊕⊕ High |
| - Medium-term (24-26 wks) | PGIC (1), UTs (1) | 0.55 [0.19, 0.91] | 327 (2) | ⊕⊕⊕⊖ Moderate^2^ |
| - Long-term | - | - | 0 (0) | No evidence |
| Function (n = 1): | | | | |
| - Immediate-term (8 wks) | 6-min walk test  Sit-to-stand test  Sit-and-reach test | 1.34 [0.32, 2.36]  1.26 [0.18, 2.34]  0.95 [-0.02, 1.91] | 19 (1)  17 (1)  19 (1) | ⊕⊖⊖⊖ Very low^6^  ⊕⊖⊖⊖ Very low^6^  ⊕⊖⊖⊖ Very low^6^ |
| - Short-term | - | - | 0 (0) | No evidence |
| - Medium-term | - | - | 0 (0) | No evidence |
| - Long-term | - | - | 0 (0) | No evidence |
| Days off work (n = 1): | | | | |
| - Immediate-term | - | - | 0 (0) | No evidence |
| - Short-term (10 wks) | % with days off work (1) | One study did not provide usable data but found no difference between groups | 168 (1) | ⊕⊖⊖⊖ Very low^6^ |
| - Medium-term | - | - | 0 (0) | No evidence |
| - Long-term | - | - | 0 (0) | No evidence |
| Imaging: no evidence | | | | |
| Physician visits: no evidence | | | | |
| Referrals: no evidence | | | | |
| Cost: no evidence | | | | |

^a^See legend on first page of S3 File for non-abbreviated names of measurement tools. **^b^**Data are presented as standardized mean differences (SMD) and 95% confidence intervals (95% CI) unless otherwise indicated (negative SMD favors education materials). Risk ratios are indicated with RR^+^ (RR > 1 favors education) and RR^-^ (RR < 1 favors education). ^c^Quality of evidence was downgraded for risk of bias,**^1^** imprecision,**^2^** inconsistency,**^3^** indirectness,**^4^** publication bias,**^5^** or downgraded to very low if there was one study.**^6^** In this comparison, downgrades for risk of bias and imprecision followed our pre-defined cut-offs (S2 File) and do not require further interpretation, and there were no downgrades for indirectness. *Due to the nature of our question (i.e., pooling the data from studies with widely varying comparator interventions), we expected considerable heterogeneity in this comparison. Therefore, if *I^2^* > 75%, we first sought to determine if the heterogeneity could be explained before downgrading the quality of evidence for inconsistency. Heterogeneity was high for short-term pain (*I^2^* = 80%), and immediate (*I^2^* = 79%) and short-term disability (*I^2^* = 85%). However, we did not downgrade for inconsistency because comparator interventions varied substantially in these comparisons, and one noticeable outlier study had a consistently larger effect in favor of the comparator intervention throughout all three of these analyses. It was a small study (n = 42) with a much higher intensity comparator intervention than all other studies (i.e., proprioceptive neuromuscular facilitation 5x/week compared to most other comparator interventions provided 1x/week). Thus, we did not downgrade for inconsistency for these comparisons because the higher intensity explains the stronger effect, the study was small and contributed little weight to the pooled estimate, and the direction of effect was the same throughout all studies in all three comparisons, so the presence or absence of this outlier is unlikely to change the result. All other comparisons with *I^2^* > 75% were downgraded for inconsistency.
