# Supplementary material for: Patient education materials for non-specific low back pain and sciatica: A systematic review and meta-analysis
Source: PLoS One. 2022 Oct 12;17(10):e0274527. doi: 10.1371/journal.pone.0274527 (PMC9555681; doi:10.1371/journal.pone.0274527)
Supplement: S4 File — (DOCX) [file pone.0274527.s004.docx]

# Forest plots for all outcomes and comparisons

**In all forest plots, the experimental group refers to patient education materials and the control group refers to the comparator (i.e., usual care or other interventions depending on the comparison).*

# Acute/subacute LBP

## Patient education materials alone vs. no intervention or usual care for acute/subacute LBP

#### Pain Intensity (n=5)


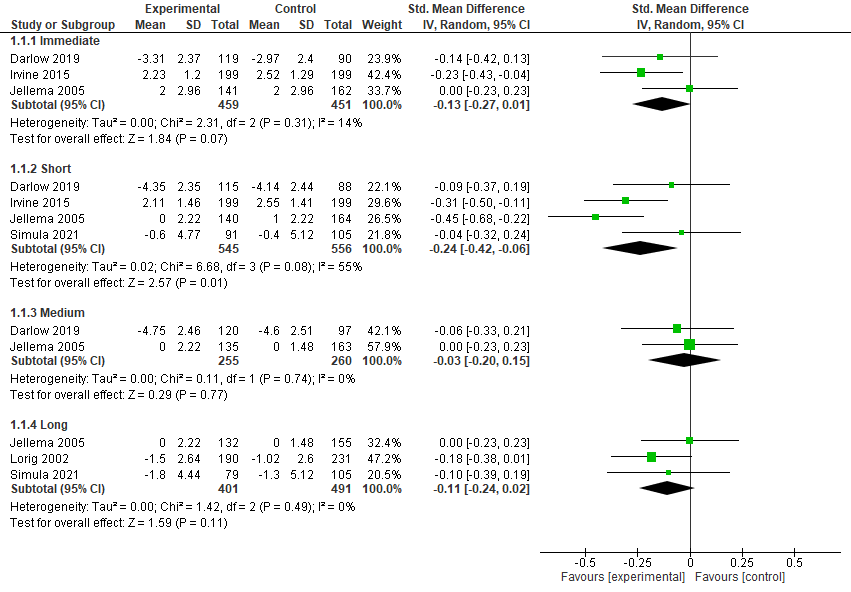


#### Disability (n=8)


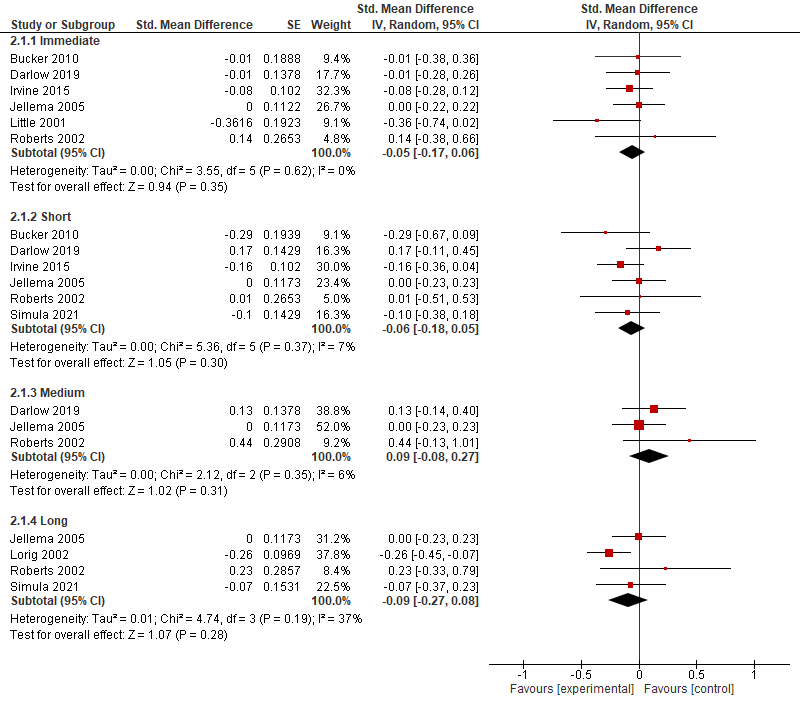


#### Quality of Life (n=4)


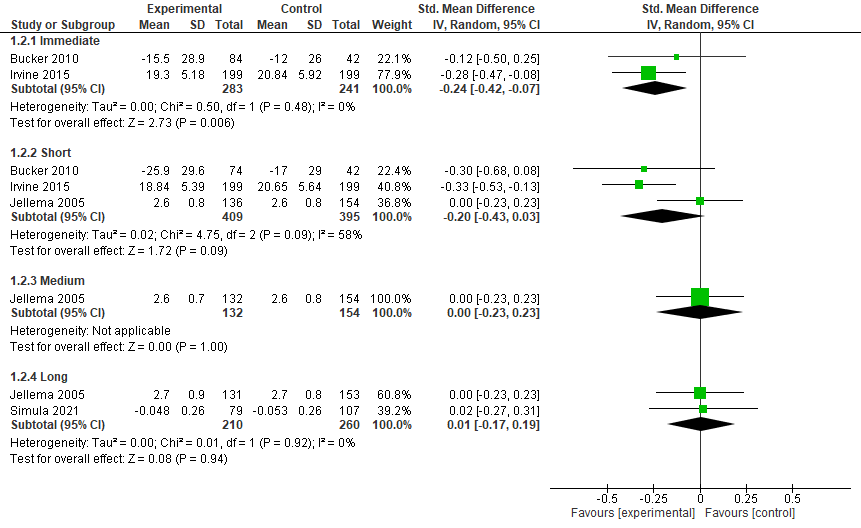


#### Global Improvement (n=1; RR < 1 favors education)


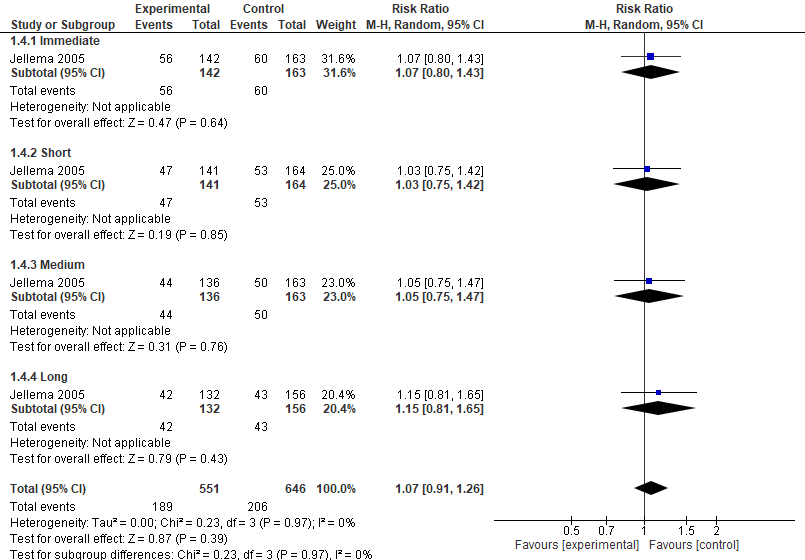


#### Knowledge (n=5; RR > 1 favors education in the long-term analysis)


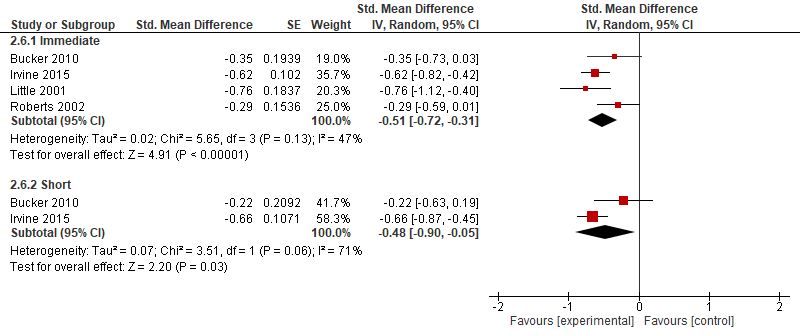


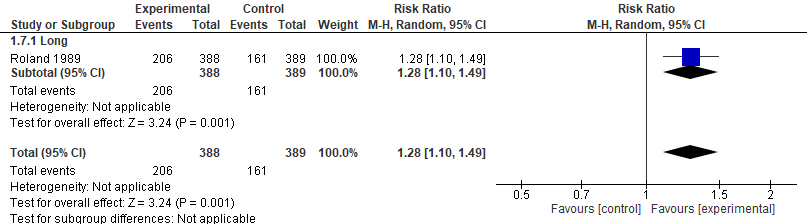


#### Pain Self-Efficacy (n=4)


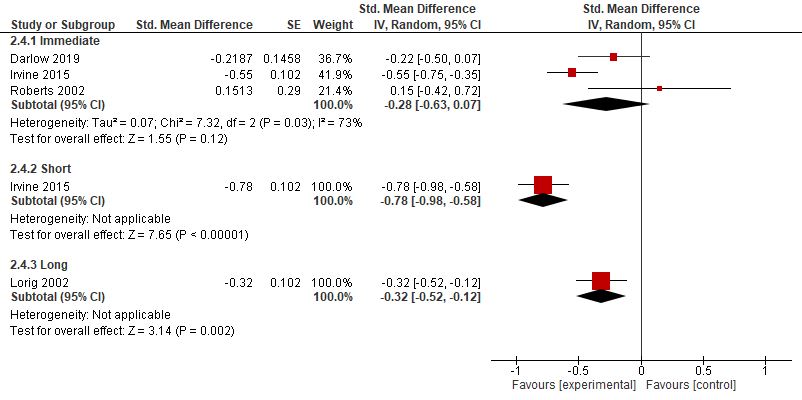


#### Fear-Avoidance Beliefs (n=3)


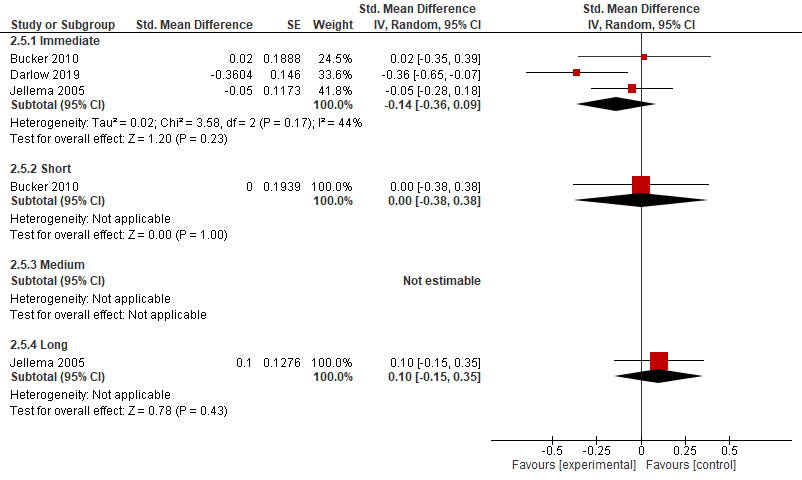
Catastrophizing (n=3)


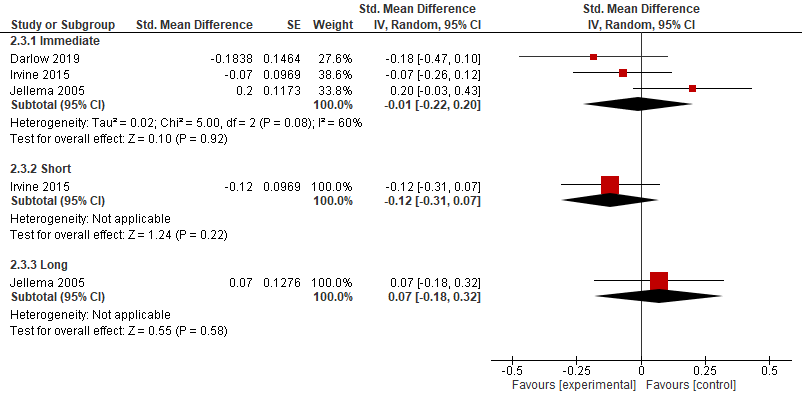


#### Anxiety (n=3)


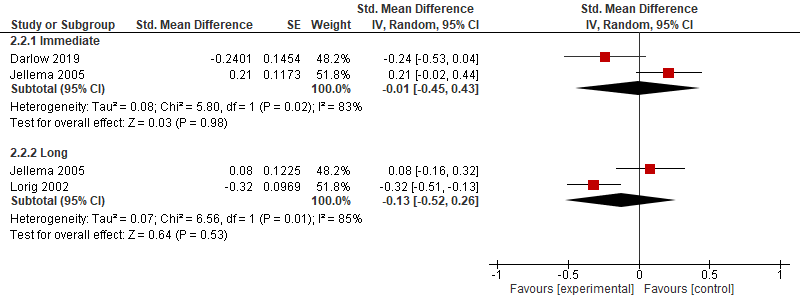


#### Days off Work (n=3; RR < 1 favors education for the immediate and medium-term analyses)


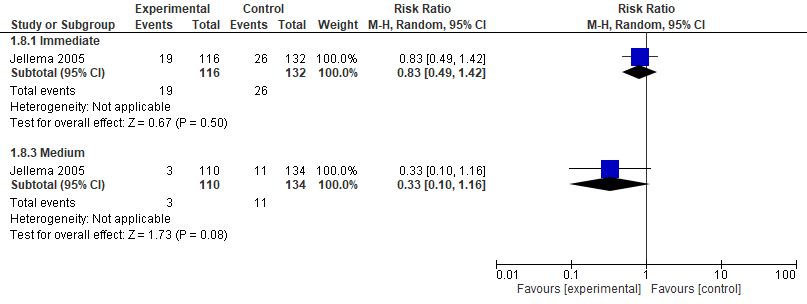


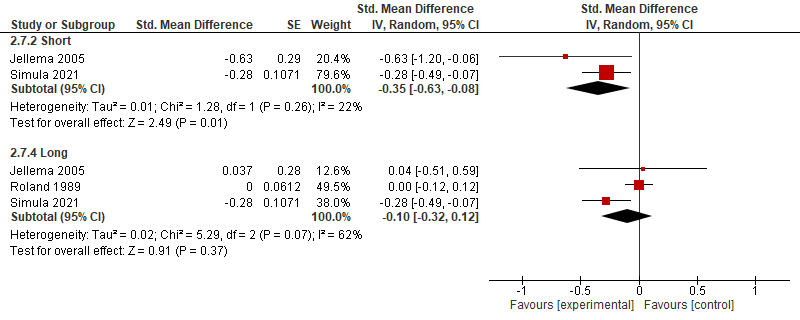


#### Imaging (n=1; RR < 1 favors education)


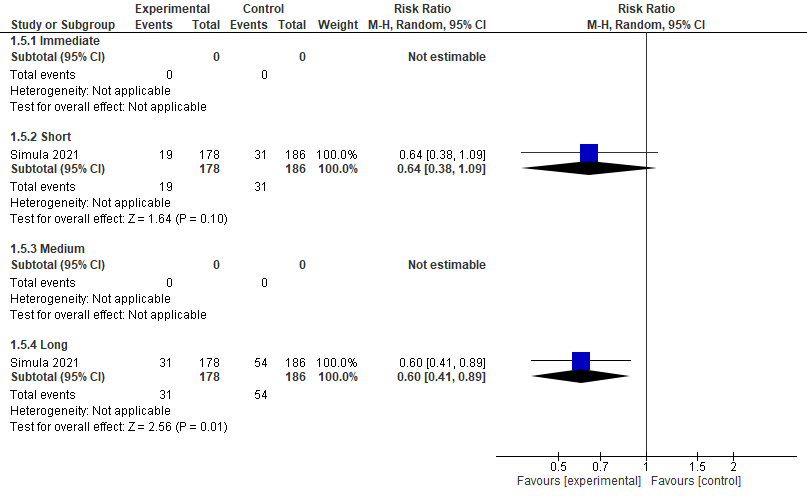


#### Physician visits (n=3)


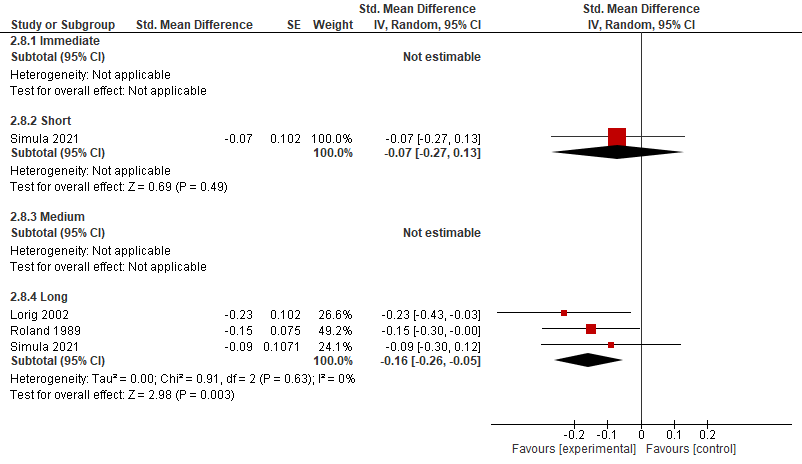


#### Referrals (n=1)


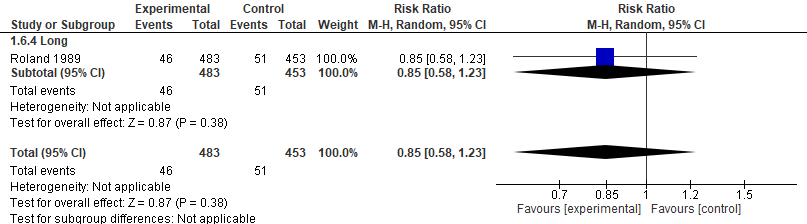


#### Cost (n=1)


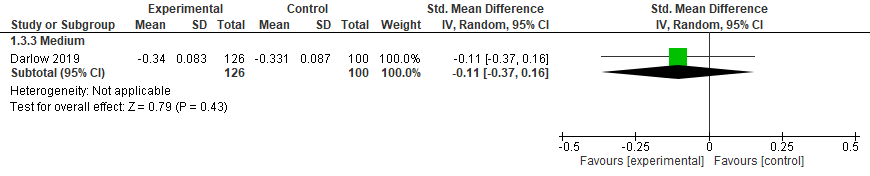


## Patient education materials alone vs. other interventions for acute/subacute LBP

### *Pain Intensity (n=3)*

*
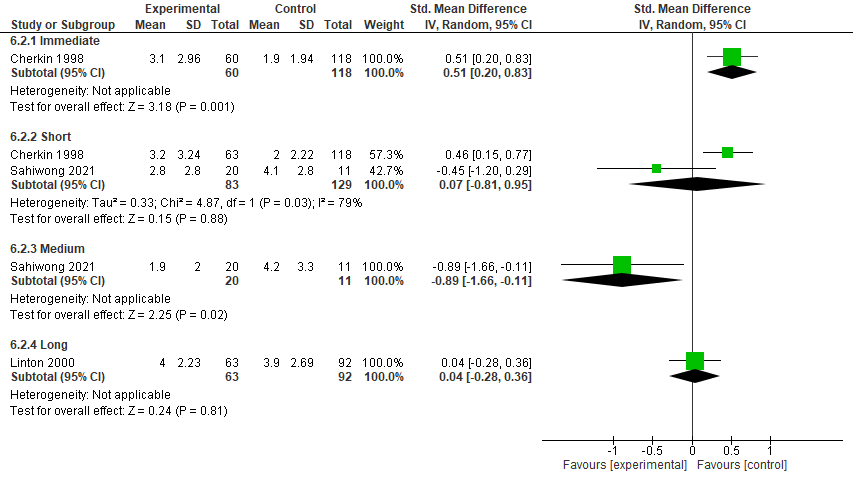
*

### *Disability (n=3)*

*
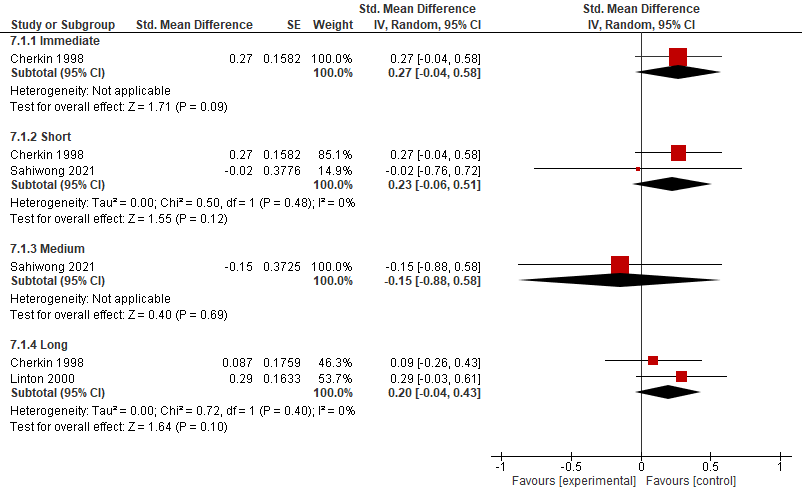
*

### *Fear-Avoidance Beliefs (n=1)*

*
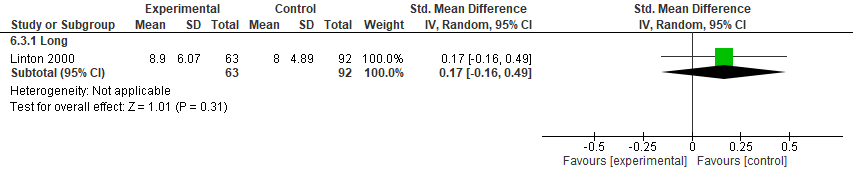
*

### *Catastrophizing (n=1)*

*
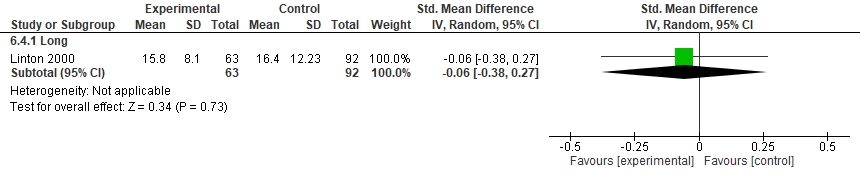
*

### *Anxiety (n=1)*

*
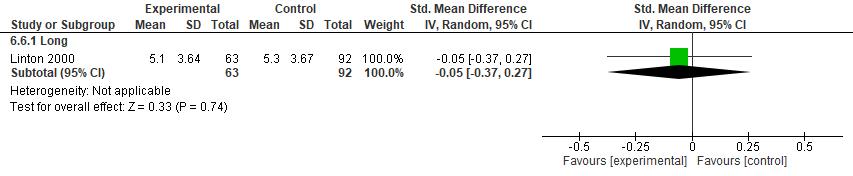
*

### *Depression (n=1)*

*
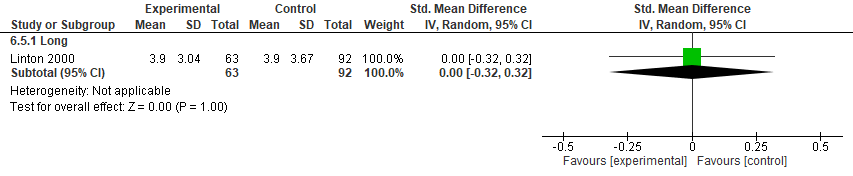
*

### *Days off Work (n=2)*

*
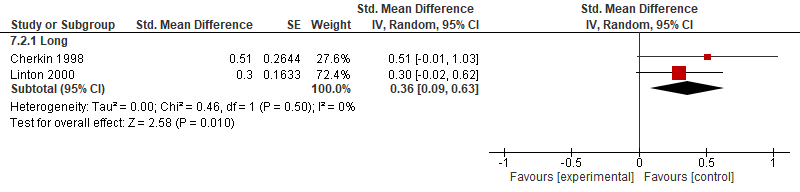
*

### *Physician Visits (n=1)*


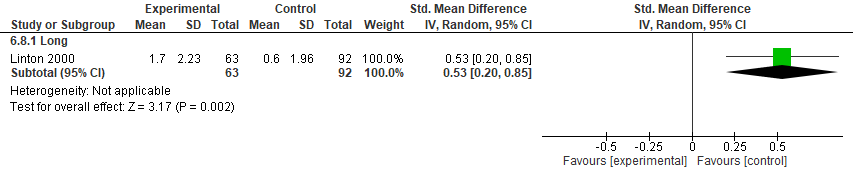


# Chronic LBP

## Patient education materials alone vs. no intervention or usual care for chronic LBP

### *Pain Intensity (n=5)*

*
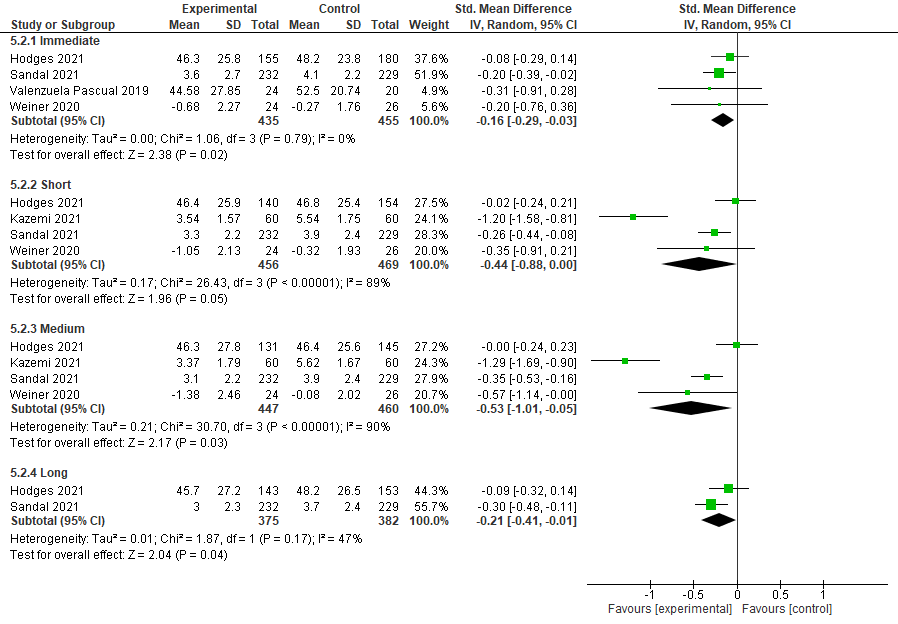
*

### *Disability (n=5)*

*
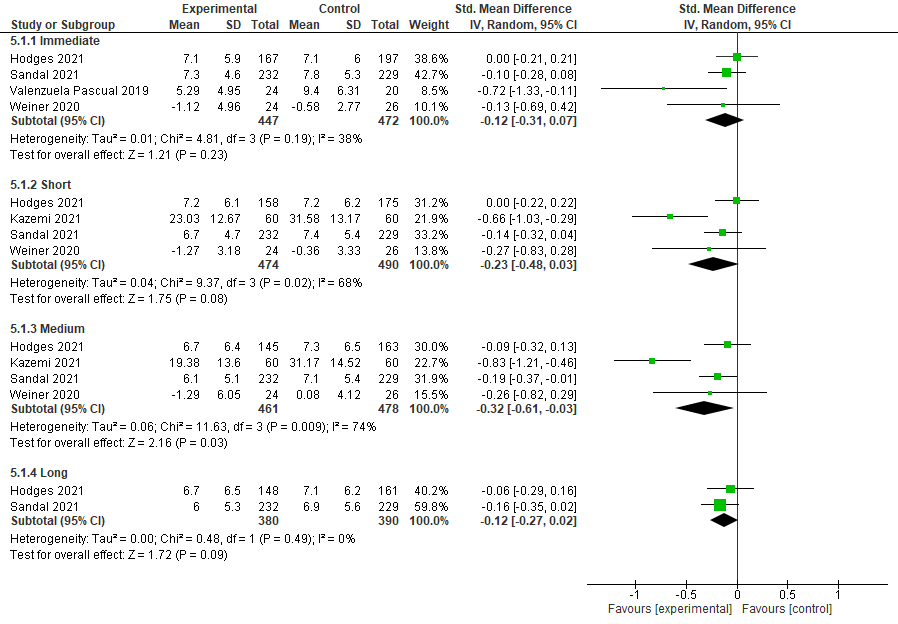
*

### *Quality of Life (n=4)*

*
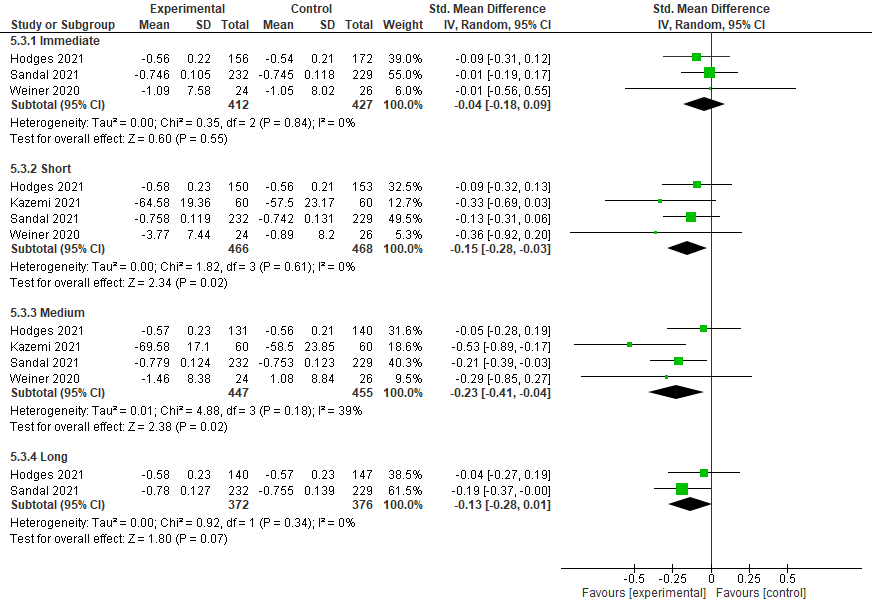
*

### *Global Improvement (n=1)*

*
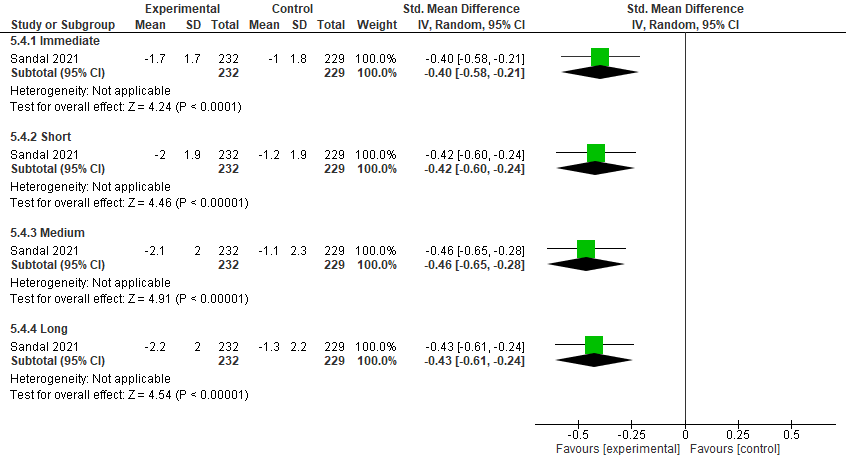
*

### *Self-efficacy (n=1)*

*
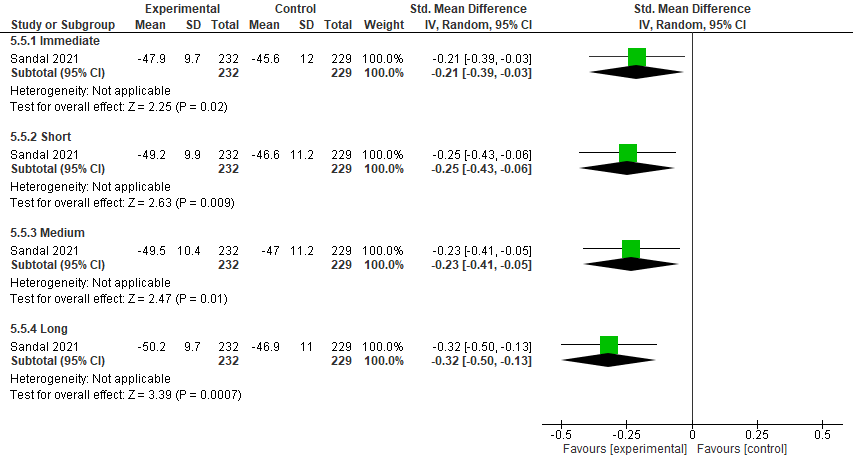
*

### *Fear-Avoidance Beliefs (n=2)*

*
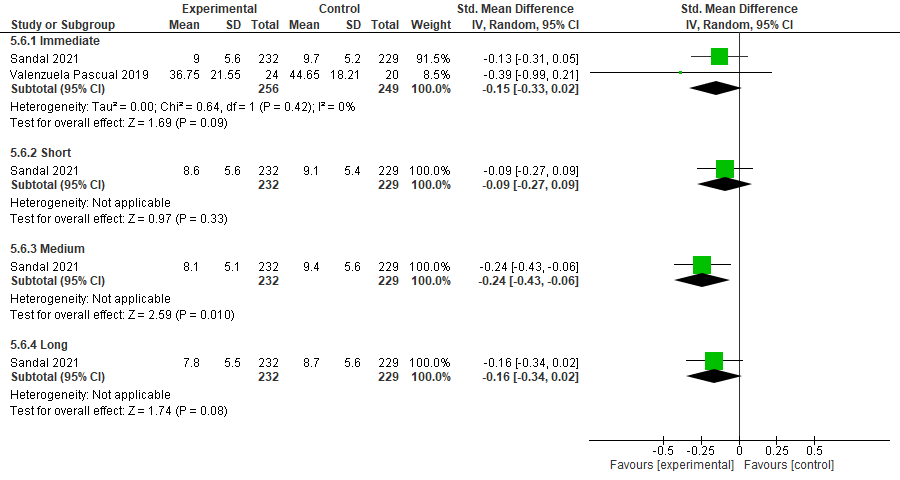
*

### *Stress (n=1)*

*
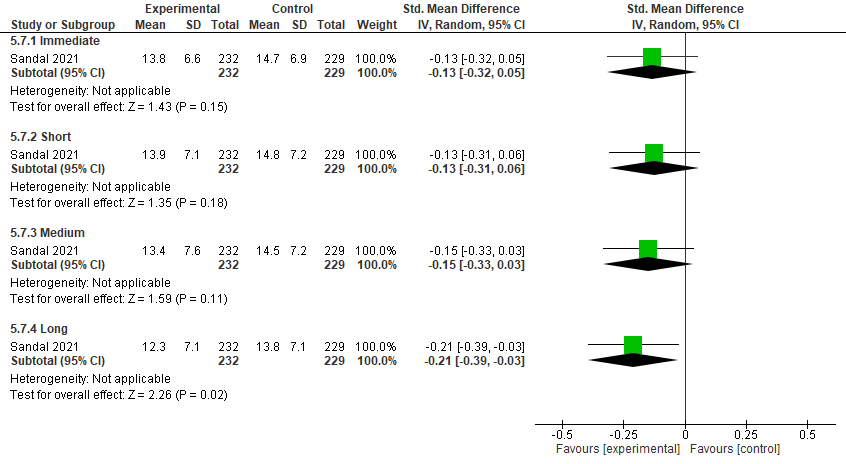
*

### *Depression (n=1)*


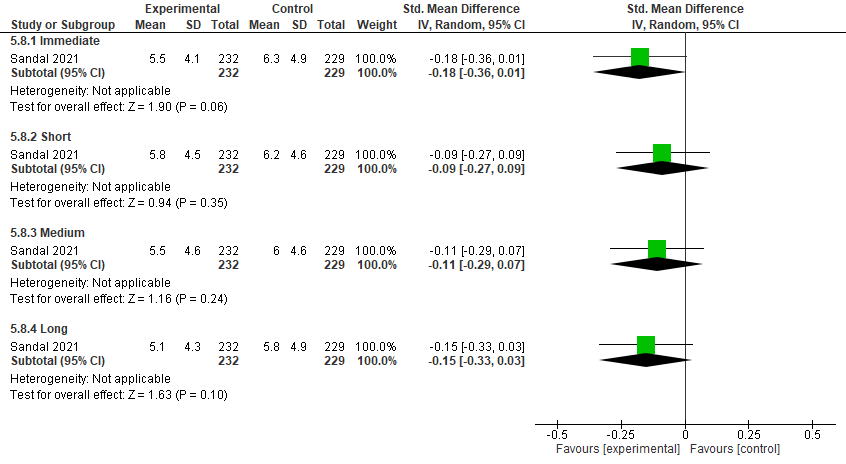


## Patient education materials alone vs. other interventions for chronic LBP

### *Pain Intensity (n=10)*

*
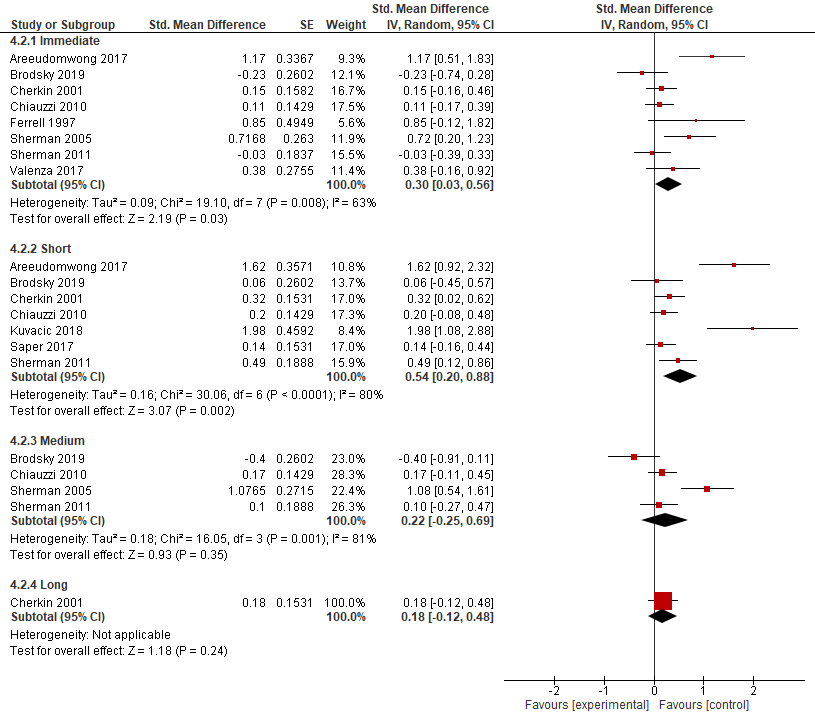
*

### *Disability (n=9)*

*
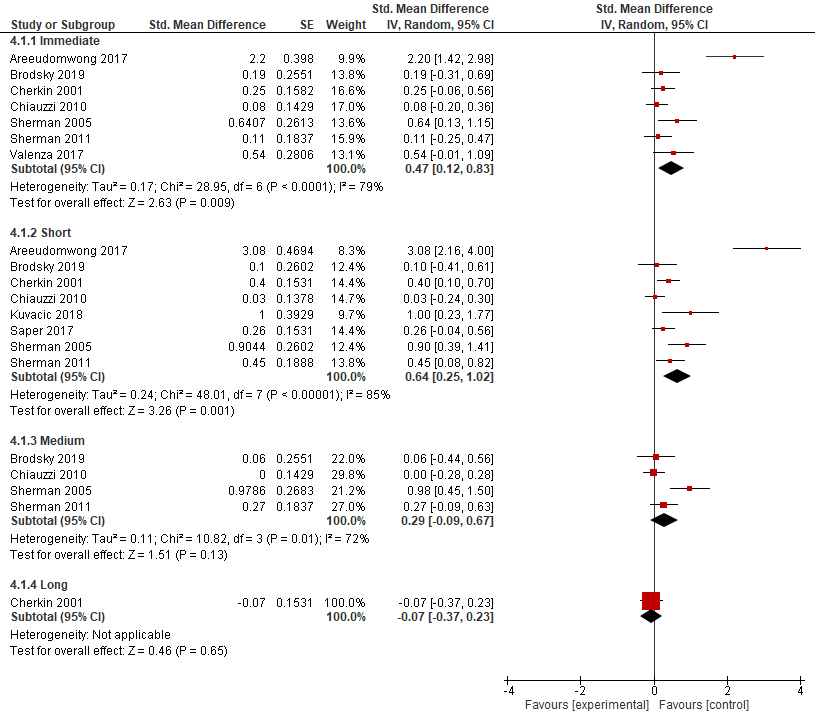
*

### *Quality of Life (n=5, however, two studies did not provide usable data and were narratively synthesized)*

*
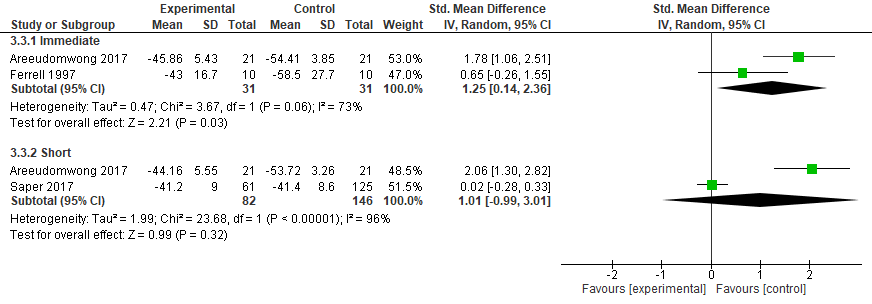
*

### *Global Improvement (n=3)*

*
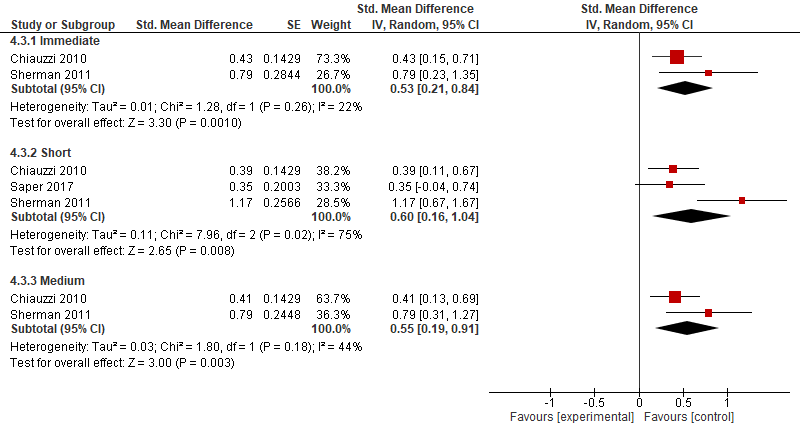
*

### *Function (n=1)*

1. 6-min. walk:

*
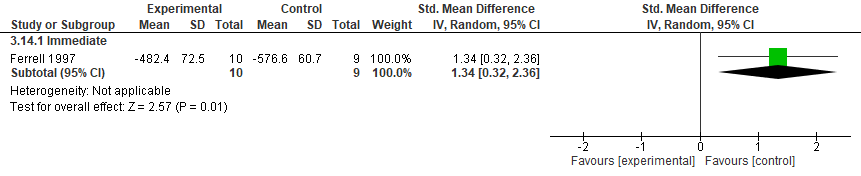
*

1. Sit-to-stand:


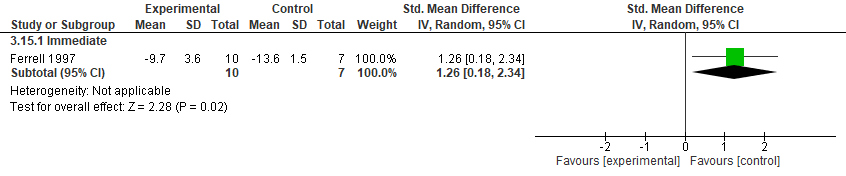


1. Sit-and-reach:


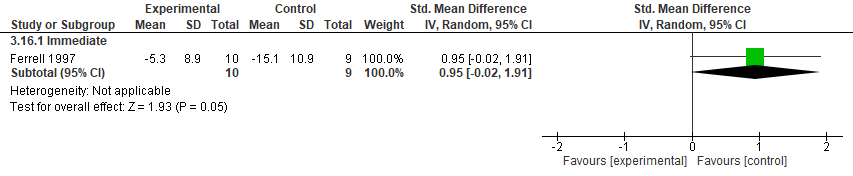


### *Pain Self-Efficacy (n=1)*

*
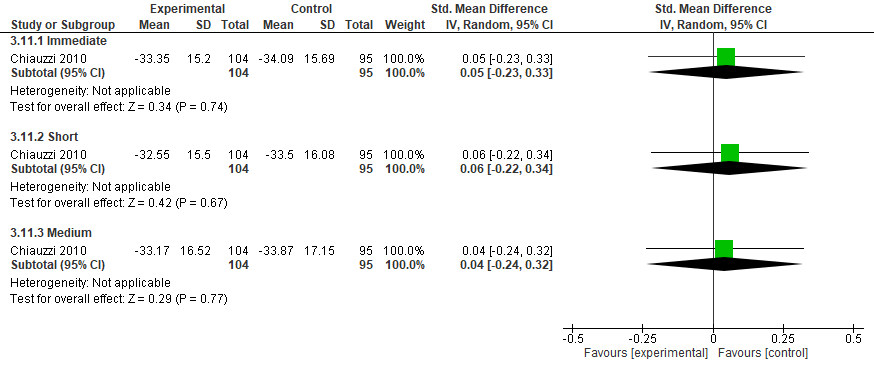
*

### *Fear-Avoidance (n=1)*

*
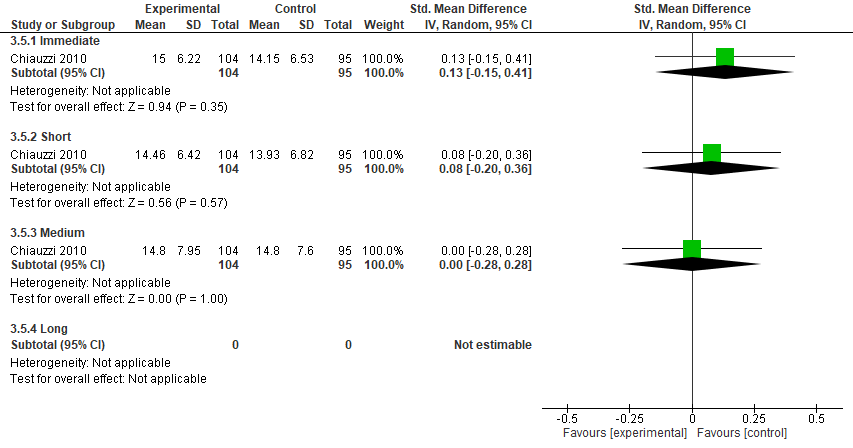
*

### *Catastrophizing (n=1)*

*
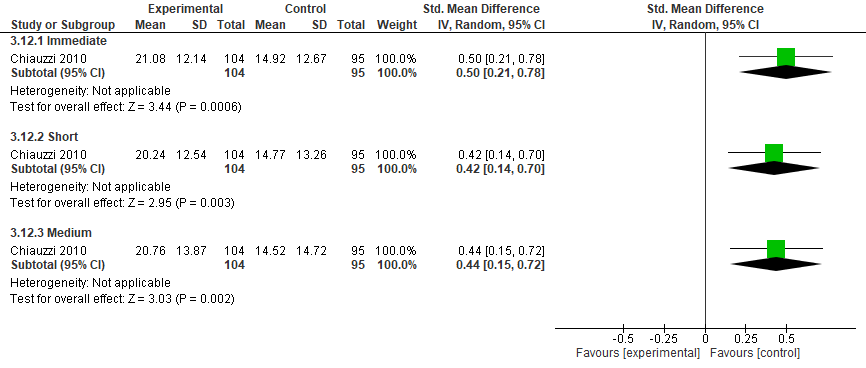
*

### *Coping (n=1)*

*
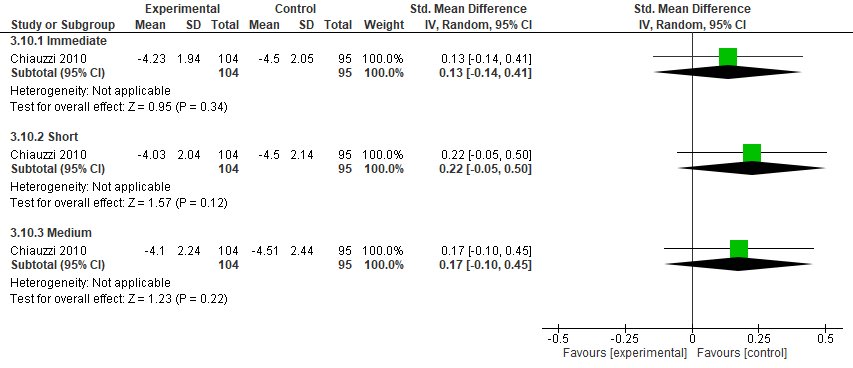
*

### *Anxiety (n=2)*

*
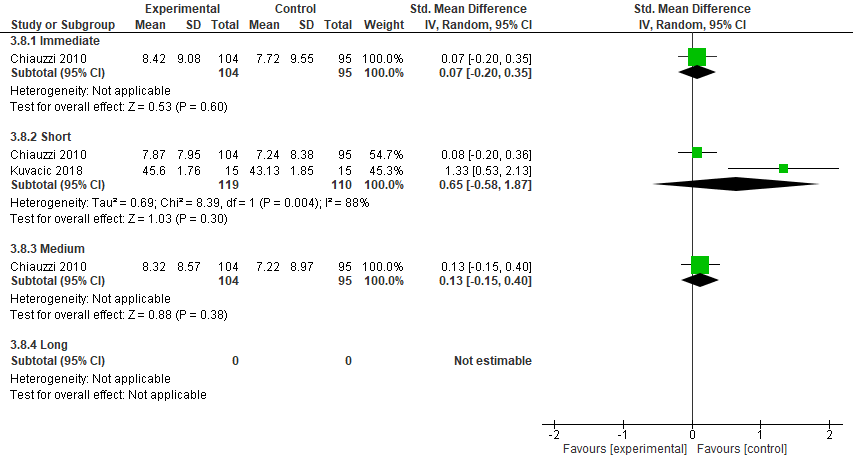
*

### *Stress (n=1)*

*
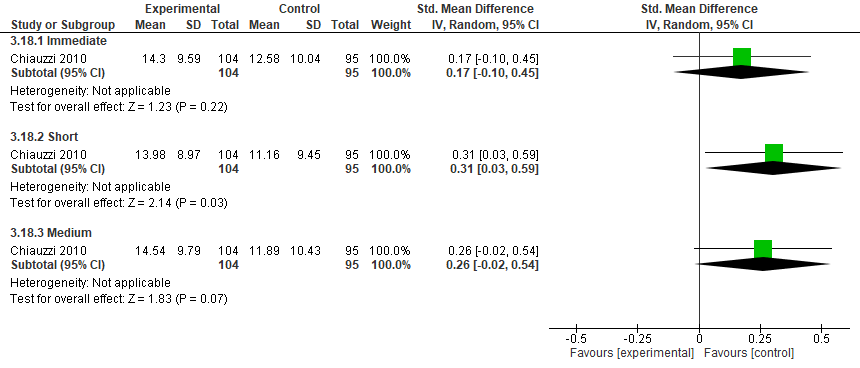
*

### *Depression (n=2)*

*
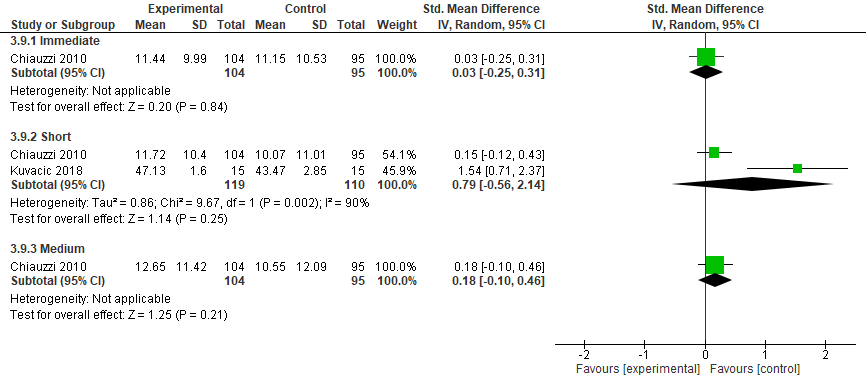
*

### *Days off Work (n=1, however, the study did not provide usable data and was narratively synthesized)*
