# Supplementary material for: Patient education materials for non-specific low back pain and sciatica: A systematic review and meta-analysis
Source: PLoS One. 2022 Oct 12;17(10):e0274527. doi: 10.1371/journal.pone.0274527 (PMC9555681; doi:10.1371/journal.pone.0274527)
Supplement: Attachment — Submitted filename: Response to Reviewers.docx [file pone.0274527.s006.docx]

**Responses to Reviewer Comments**

Reviewer #1: The authors presented a meta-analysis of 27 studies evaluating patient education materials for non-specific low back pain and sciatica. The analysis is well-conducted, data is presented in an intelligible manner, and the manuscript has been drafted decently. However, there are a few minor concerns that need to be addressed.

**1. In the abstract, the authors have mentioned the results but the relevant numbers are not provided within brackets. Please include the effect sizes within brackets in the abstract as well.**

We agree that adding the effect sizes would add value to the abstract and have now added all the relevant numbers (pg. 2, line 26). Please note that this did require us to exceed PLOS One’s word limit for abstracts. **2. In the data extraction section, kindly mention the initials of the reviewers within brackets who have conducted the data abstraction. Also add as to what was done in case there were any discrepancies during screening articles or data extraction. Was a third reviewer consulted or the issues were solved mutually? Please add this too.**

Please note that all data abstraction was performed by BF and doubly abstracted by one of three individuals who have been acknowledged in the acknowledgements section (GD, AS, SG). These contributors only partially contributed to portions of study screening and/or data extraction and do not qualify for authorship. However, we have still added their initials in these sections as requested with reference to the acknowledgements section.

Thank you for your second point. We had already described how screening discrepancies/conflicts would be resolved (i.e., by a third reviewer; please see pg. 5, line 101), but did not do so for the data extraction section. We have now included this (pg. 6, line 108) with the author initials in all sections. **3. Review Manager 5 was used for statistical analysis. Can the authors please add the company details of the software within brackets?**

We have added the specific version (5.4.1) and company (The Cochrane Collaboration) details for Review Manager 5 in the manuscript (pg. 7, line 139). **4. Kindly cite the reference for Higgins I2 (square) statistic.**

We have cited this paper for the *I^2^* statistic (pg. 8, line 151)**.

5. In the results section, the authors have mentioned the effect sizes and number of RCTs and patients within brackets. However, two important components of meta-analytical results are missing. Kindly add the p values and I2 (square) values within all these brackets as well.**

Thank you for pointing this out, we agree that this will add value to the results section. We have added all relevant *p* and *I^2^* values to the text (starting on pg. 21, line 227).
