# Supplementary material for: Patient education materials for non-specific low back pain and sciatica: A systematic review and meta-analysis
Source: PLoS One. 2022 Oct 12;17(10):e0274527. doi: 10.1371/journal.pone.0274527 (PMC9555681; doi:10.1371/journal.pone.0274527)
Supplement: Attachment — Submitted filename: Response to Reviewers.docx [file pone.0274527.s007.docx]

**Responses to Reviewer Comments**

Reviewer #1: The authors have addressed the concerns appropriately. However, there is a minor issue. The authors state, 'Two reviewers (BF, see acknowledgements for AS, SG)'. However, these are a total of 3 reviewers I suppose. Kindly correct this if it is an error.

Thank you very much for noticing the wording here. We completely agree that the way we worded this could be confusing for the reader. What we meant to say is that BF performed all actions for all studies, and it was doubly performed by *one of* the other listed reviewers (e.g., *either* GD, AS, SG). That is, the work was split up between these three acknowledged individuals so that all screening and data extraction were performed by two reviewers. To clarify, if we had a hypothetical scenario where 1000 studies were found in the search, BF would have screened all 1000 articles, and GD, AS, and SG would have each screened approximately 333 unique articles so that all articles were screened by two reviewers in the end. I hope this makes sense.

We have updated the wording in these instances to make it more explicitly clear:

- pg. 5, line 100 – changed to “(BF, one of GD, AS, SG; see acknowledgements)”
- pg. 6, line 107 – changed to “(BF, one of AS, SG; see acknowledgements)”
